# Crystal structure of benzyl 2-naphthyl ether, a sensitiser for thermal paper

**DOI:** 10.1107/S2056989019000690

**Published:** 2019-01-18

**Authors:** Takuya Kikuchi, Saori Gontani, Kyohei Miyanaga, Takaaki Kurata, Yoshiki Akatani, Shinya Matsumoto

**Affiliations:** a Graduate School of Environment and Information Sciences, Yokohama National University, Tokiwadai 79-7, Hodogaya-ku, Yokohama 240-8501, Japan; b Functional Chemicals R&D Laboratories, Nippon Kayaku Corporation Limited, Shimo 3-31-2, Kita-ku, Tokyo 115-8588, Japan; c Color Materials Division in Functional Chemicals Group, Nippon Kayaku Corporation Limited, Shimo 3-31-2, Kita-ku, Tokyo 115-8588, Japan

**Keywords:** crystal structure, thermal paper, sensitiser, C—H⋯π inter­molecular inter­action

## Abstract

The title compound [systematic name: 2-(benz­yloxy)naphthalene], C_17_H_14_O, which is used as a sensitiser for thermal paper, has a twisted conformation with a dihedral angle of 48.71 (12)° between the phenyl ring and the naphthyl ring system. In the crystal, one mol­ecule inter­acts with six neighbouring mol­ecules *via* inter­molecular C—H⋯π inter­actions to form a herringbone mol­ecular arrangement.

## Abstract 2

We report the crystal structure of benzyl 2-naphthyl ether, which is used as a sensitiser for thermal paper. In the crystal, one mol­ecule inter­acts with six neighbouring mol­ecules *via* C—H⋯π inter­molecular inter­actions to form a herringbone mol­ecular arrangement.

## Chemical context   

Thermal printing is a rapid and inexpensive printing technology widely used in commercial applications such as receipts, faxes and tickets (Gregory, 1991 ▸; Mendum *et al.*, 2011 ▸). Many structural reports are available for thermosensitive dyes and developers (Matsumoto *et al.*, 2010 ▸; Kodama *et al.*, 2013 ▸; Gontani *et al.*, 2017 ▸; Ohashi *et al.*, 2017 ▸). On the other hand, we found only one report on the crystal structure of a compound commonly used as a sensitiser for the thermosensitive layer (Rudolph *et al.*, 2010 ▸), which can facilitate the dye coloration process by lowering the melting point of the dye/developer composite on thermal paper (US EPA, 2014 ▸). The title compound, benzyl 2-naphthyl ether, **1**, is known as another commonly used sensitiser. Herein, we report the crystal structure of **1** as fundamental data for the investigation of its influence on the solid-state physicochemical properties of the thermosensitive layer of the thermal paper.
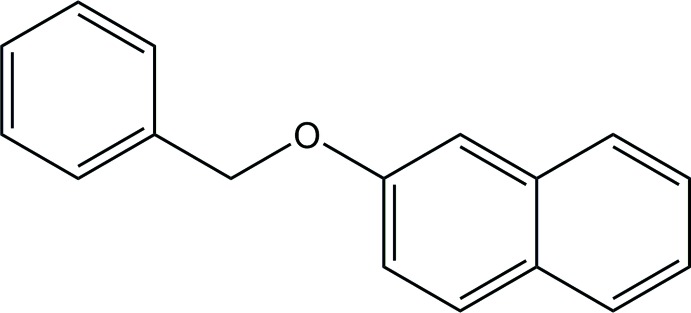


## Structural commentary   

**Figure 1:**
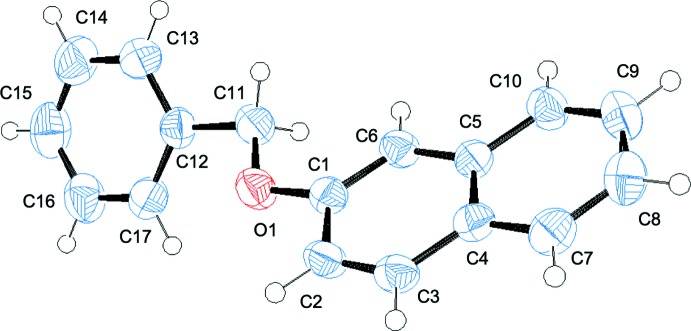
The mol­ecular structure of the title compound, **1**, with displacement ellipsoids drawn at the 50% probability level.

The title compound (Fig. 1 ▸) is a simple ether compound in which a benzyl group is connected to a naphthyl group *via* an ether bond. The two aromatic rings are twisted, which is mainly attributable to the rotation about the C11—C12 bond. The dihedral angle between the mean planes of the naphthyl ring system (C1–C10) and the phenyl ring (C12–C17) is 48.71 (12)°. The related torsion angles for this dihedral angle are −44.9 (3)° (O1—C11—C12—C17), 178.7 (2)° (C1—O1—C11—C12) and −5.6 (3)° (C6—C1—O1—C11).

## Supra­molecular features   

**Figure 2:**
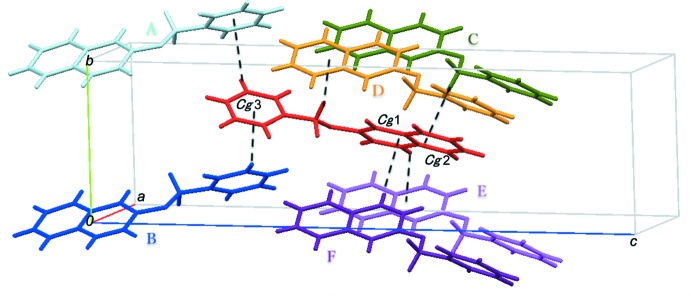
A packing diagram of the title compound, **1**, showing inter­molecular C—H⋯π inter­actions (dashed lines). [Symmetry codes: (A) −*x* + 1, *y* + 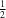, −*z* + 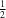; (B) −*x* + 1, *y* − 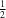, −*z* + 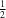; (C) *x* + 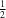, −*y* + 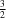, −*z* + 1; (D) *x* − 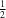, −*y* + 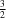, −*z* + 1; (E) *x* + 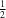, −*y* + 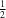, −*z* + 1; (F) *x* − 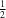, −*y* + 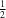, −*z* + 1.]

**Figure 3:**
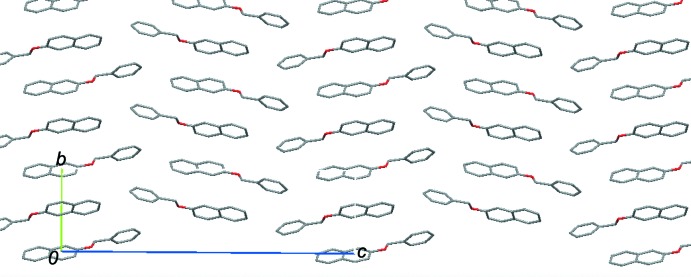
A packing diagram of the title compound, **1**, viewed along the *a* axis, showing a herringbone arrangement. H atoms have been omitted for clarity.

In the crystal, one mol­ecule inter­acts with six neighbouring mol­ecules *via* inter­molecular C—H⋯π inter­actions (Table 1 ▸; Fig. 2 ▸). The mol­ecules are linked by a C—H⋯π inter­action between the benzene C1–C6 rings (C3—H3⋯*Cg*1^i^; symmetry code as in Table 1 ▸), forming a zigzag chain along the *a*-axis direction. The chains are connected into a layer structure parallel to the *ab* plane *via* a C—H⋯π inter­action between the benzene C4/C5/C7–C10 ring and the methyl­ene hydrogen atom (C11—H11*A*⋯*Cg*2^ii^; Table 1 ▸). A weak C—H⋯π inter­action between the C12–C17 phenyl rings (C16—H16⋯*Cg*3^iii^; Table 1 ▸) links the layers and thus the mol­ecules form a herringbone arrangement when viewed along the *a* axis, as shown in Fig. 3 ▸.

## Database survey   

**Figure 4:**
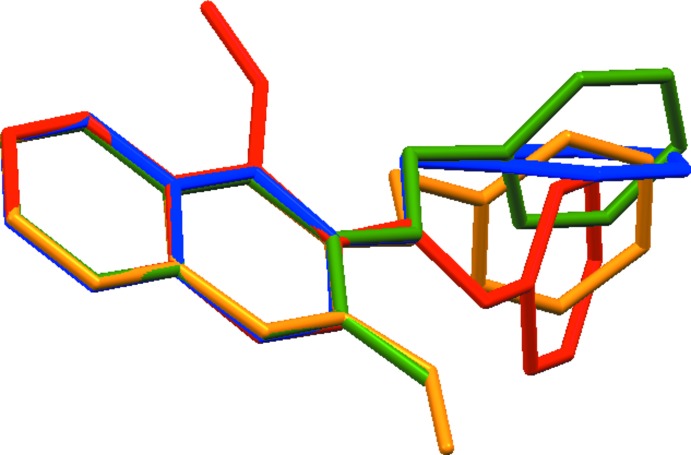
An overlay of the mol­ecular conformation of four analogous benzyl-2-naphthyl ether derivatives, **1** (blue), **2** (red), **3** (yellow) and **4** (green). All H atoms have been omitted for clarity.

**Figure 5:**
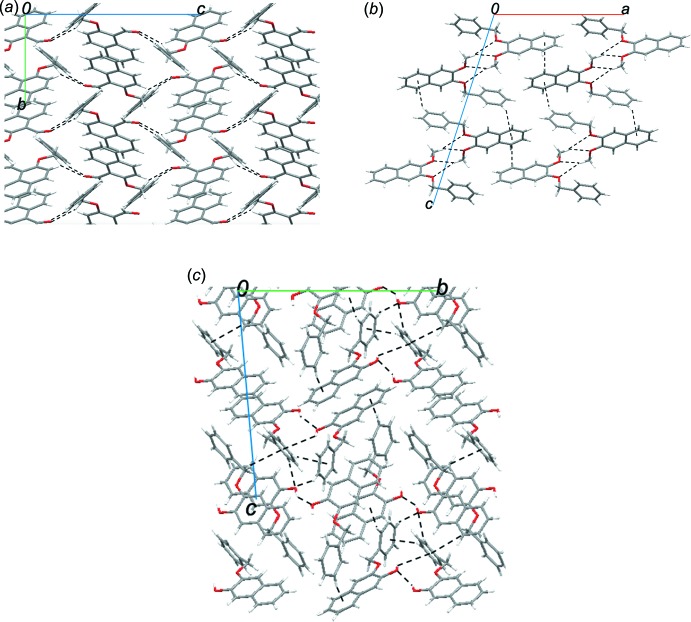
Packing diagrams of compounds **2** (*a*), **3** (*b*) and **4** (*c*). The dotted lines indicate inter­molecular C—H⋯O and C—H⋯π inter­actions.

Three analogous compounds of **1**, namely, 2-benz­yloxy-1-naphthaldehyde, **2** [CSD (Groom *et al.*, 2016 ▸) refcode SOLVUL; Gao *et al.*, 2009 ▸], 2-benz­yloxy-3-meth­oxy­naphthalene, **3** (MEBYIC; Huang *et al.*, 2004 ▸) and 2-benz­yloxy-3-hy­droxy­naphthalene, **4** (SICGEQ; Peters *et al.*, 1998 ▸), have been reported. Compounds, **2**, **3** and **4**, crystallize in the centrosymmetric space groups *P*2_1_/*c*, *P*2_1_/*c* and *P* 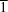, respectively. Fig. 4 ▸ shows an overlay of the mol­ecular geometries of compounds **1**–**4**, which indicates significant geometrical differences in the conformation of the benzyl unit caused by the rotations around the C1—O1 and C11—C12 bonds. Fig. 5 ▸ shows packing diagrams for compounds **2**–**4**. In the crystals of **2**–**4**, the mol­ecules form zigzag chains *via* C—H⋯O inter­actions. In **2**, the chains are linked by π–π inter­actions into a three-dimensional network, whereas C—H⋯π inter­actions contribute to the arrangement of the chains in **3** and **4**.

## Synthesis and crystallization   

The title compound was purchased from Tokyo Kasei Kogyo Co., Ltd., and used without further purification. X-ray diffraction quality colourless platelets were obtained using a liquid–liquid diffusion method, with combination of chloro­form and ethanol at 278 K.

## Refinement   

The crystal data, data collection and structure refinement details are summarized in Table 2 ▸. All H atoms were positioned geometrically (C—H = 0.93 Å) and refined using a riding model with *U*_iso_(H) = 1.2*U*_eq_(C).

## Supplementary Material

Crystal structure: contains datablock(s) global, I. DOI: 10.1107/S2056989019000690/is5508sup1.cif

Structure factors: contains datablock(s) I. DOI: 10.1107/S2056989019000690/is5508Isup2.hkl

Supporting information file. DOI: 10.1107/S2056989019000690/is5508Isup3.cml

CCDC reference: 1890872

Additional supporting information:  crystallographic information; 3D view; checkCIF report

## Figures and Tables

**Table 1:** Hydrogen-bond geometry (Å, °) *Cg*1, *Cg*2 and *Cg*3 are the centroids of the C1–C6, C4/C5/C7–C10 and C12–C17 rings, respectively.

*D*—H⋯*A*	*D*—H	H⋯*A*	*D*⋯*A*	*D*—H⋯*A*
C3—H3⋯*Cg*1^i^	0.93	2.71	3.439 (2)	135
C11—H11*A*⋯*Cg*2^ii^	0.97	2.63	3.512 (3)	150
C16—H16⋯*Cg*3^iii^	0.93	2.87	3.586 (3)	135

Symmetry codes: (i) 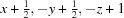; (ii) 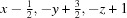; (iii) 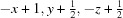.

**Table 2:** Experimental details

Crystal data	
Chemical formula	C_17_H_14_O
*M*_r_	234.30
Crystal system, space group	Orthorhombic, *P*2_1_2_1_2_1_
Temperature (K)	298
*a*, *b*, *c* (Å)	6.10537 (10), 7.58687 (13), 26.8196 (5)
*V* (Å^3^)	1242.30 (4)
*Z*	4
Radiation type	Cu *K*α
μ (mm^−1^)	0.59
Crystal size (mm)	0.61 × 0.42 × 0.04

Data collection	
Diffractometer	Rigaku XtaLAB PRO
Absorption correction	Multi-scan (*CrysAlis PRO*; Rigaku OD, 2015 ▸)
*T*_min_, *T*_max_	0.396, 0.976
No. of measured, independent and observed [*F* ^2^ > 2.0σ(*F* ^2^)] reflections	3724, 2017, 1841
*R*_int_	0.033
(sin θ/λ)_max_ (Å^−1^)	0.594

Refinement	
*R*[*F* ^2^ > 2σ(*F* ^2^)], *wR*(*F* ^2^), *S*	0.042, 0.107, 1.01
No. of reflections	2017
No. of parameters	163
H-atom treatment	H-atom parameters constrained
Δρ_max_, Δρ_min_ (e Å^−3^)	0.12, −0.20
Absolute structure	Flack *x* determined using 601 quotients [(*I* ^+^)−(*I* ^−^)]/[(*I* ^+^)+(*I* ^−^)] (Parsons *et al.*, 2013 ▸)
Absolute structure parameter	−0.3 (3)

Computer programs: *CrysAlis PRO* (Rigaku OD, 2015 ▸), *SHELXT* (Sheldrick, 2015*a* ▸), *SHELXL2014* (Sheldrick, 2015*b* ▸) and *CrystalStructure* (Rigaku, 2018 ▸).

## supplementary crystallographic information

### Crystal data

**Table:**

C_17_H_14_O	*D*_x_ = 1.253 Mg m^−^^3^
*M**_r_* = 234.30	Cu *K*α radiation, λ = 1.54184 Å
Orthorhombic, *P*2_1_2_1_2_1_	Cell parameters from 2523 reflections
*a* = 6.10537 (10) Å	θ = 6.1–71.1°
*b* = 7.58687 (13) Å	µ = 0.59 mm^−^^1^
*c* = 26.8196 (5) Å	*T* = 298 K
*V* = 1242.30 (4) Å^3^	Plate, colourless
*Z* = 4	0.61 × 0.42 × 0.04 mm
*F*(000) = 496.00	

### Data collection

**Table:**

Rigaku XtaLAB PRO diffractometer	1841 reflections with *F*^2^ > 2.0σ(*F*^2^)
Detector resolution: 5.811 pixels mm^-1^	*R*_int_ = 0.033
ω scans	θ_max_ = 66.4°, θ_min_ = 3.3°
Absorption correction: multi-scan (CrysAlis PRO; Rigaku OD, 2015)	*h* = −3→7
*T*_min_ = 0.396, *T*_max_ = 0.976	*k* = −9→8
3724 measured reflections	*l* = −31→31
2017 independent reflections	

### Refinement

**Table:**

Refinement on *F*^2^	Hydrogen site location: inferred from neighbouring sites
*R*[*F*^2^ > 2σ(*F*^2^)] = 0.042	H-atom parameters constrained
*wR*(*F*^2^) = 0.107	*w* = 1/[σ^2^(*F*_o_^2^) + (0.0559*P*)^2^] where *P* = (*F*_o_^2^ + 2*F*_c_^2^)/3
*S* = 1.01	(Δ/σ)_max_ < 0.001
2017 reflections	Δρ_max_ = 0.12 e Å^−^^3^
163 parameters	Δρ_min_ = −0.20 e Å^−^^3^
0 restraints	Absolute structure: Flack *x* determined using 601 quotients [(*I*^+^)-(*I*^-^)]/[(*I*^+^)+(*I*^-^)] (Parsons *et al*., 2013)
Primary atom site location: structure-invariant direct methods	Absolute structure parameter: −0.3 (3)
Secondary atom site location: difference Fourier map	

### Special details

**Table:**

Geometry. All esds (except the esd in the dihedral angle between two l.s. planes) are estimated using the full covariance matrix. The cell esds are taken into account individually in the estimation of esds in distances, angles and torsion angles; correlations between esds in cell parameters are only used when they are defined by crystal symmetry. An approximate (isotropic) treatment of cell esds is used for estimating esds involving l.s. planes.
Refinement. Refinement was performed using all reflections. The weighted R-factor (wR) and goodness of fit (S) are based on F^2^. R-factor (gt) are based on F. The threshold expression of F^2^ > 2.0 sigma(F^2^) is used only for calculating R-factor (gt).

### Fractional atomic coordinates and isotropic or equivalent isotropic displacement parameters (Å^2^)

**Table:**

	*x*	*y*	*z*	*U*_iso_*/*U*_eq_	
O1	0.5853 (3)	0.5557 (2)	0.39703 (5)	0.0492 (4)	
C1	0.6358 (4)	0.5250 (3)	0.44606 (8)	0.0394 (5)	
C5	0.5738 (4)	0.5198 (3)	0.53498 (7)	0.0380 (5)	
C3	0.9115 (3)	0.4027 (3)	0.50010 (8)	0.0432 (5)	
H3	1.0488	0.3518	0.5045	0.052*	
C4	0.7789 (4)	0.4363 (3)	0.54226 (8)	0.0393 (5)	
C12	0.3435 (4)	0.6519 (3)	0.33234 (8)	0.0446 (5)	
C6	0.5058 (3)	0.5652 (3)	0.48601 (8)	0.0405 (5)	
H6	0.3728	0.6223	0.4811	0.049*	
C7	0.8415 (4)	0.3848 (3)	0.59097 (9)	0.0494 (6)	
H7	0.9760	0.3300	0.5960	0.059*	
C2	0.8426 (4)	0.4432 (3)	0.45349 (9)	0.0447 (5)	
H2	0.9310	0.4173	0.4262	0.054*	
C10	0.4399 (4)	0.5505 (3)	0.57705 (8)	0.0466 (5)	
H10	0.3060	0.6072	0.5731	0.056*	
C11	0.3713 (4)	0.6215 (4)	0.38720 (8)	0.0491 (6)	
H11A	0.3492	0.7313	0.4051	0.059*	
H11B	0.2628	0.5374	0.3987	0.059*	
C8	0.7069 (5)	0.4148 (4)	0.63052 (9)	0.0561 (6)	
H8	0.7496	0.3798	0.6623	0.067*	
C9	0.5047 (5)	0.4980 (3)	0.62363 (9)	0.0547 (6)	
H9	0.4136	0.5177	0.6509	0.066*	
C13	0.1486 (4)	0.6077 (4)	0.30972 (9)	0.0572 (6)	
H13	0.0402	0.5509	0.3280	0.069*	
C17	0.5030 (5)	0.7337 (4)	0.30452 (9)	0.0557 (6)	
H17	0.6365	0.7617	0.3192	0.067*	
C16	0.4669 (6)	0.7750 (4)	0.25472 (10)	0.0650 (8)	
H16	0.5752	0.8315	0.2363	0.078*	
C14	0.1122 (5)	0.6472 (5)	0.25993 (10)	0.0721 (9)	
H14	−0.0195	0.6162	0.2449	0.086*	
C15	0.2717 (5)	0.7322 (4)	0.23289 (10)	0.0706 (9)	
H15	0.2467	0.7607	0.1996	0.085*	

### Atomic displacement parameters (Å^2^)

**Table:**

	*U*^11^	*U*^22^	*U*^33^	*U*^12^	*U*^13^	*U*^23^
O1	0.0436 (8)	0.0647 (10)	0.0394 (8)	0.0042 (7)	0.0028 (7)	0.0050 (7)
C1	0.0388 (11)	0.0405 (11)	0.0387 (11)	−0.0026 (9)	0.0009 (9)	0.0015 (8)
C5	0.0394 (11)	0.0352 (10)	0.0393 (11)	−0.0026 (8)	0.0009 (9)	−0.0017 (9)
C3	0.0325 (9)	0.0461 (11)	0.0510 (13)	0.0024 (9)	0.0007 (11)	−0.0012 (10)
C4	0.0366 (10)	0.0367 (10)	0.0448 (11)	−0.0015 (9)	−0.0019 (10)	−0.0017 (9)
C12	0.0473 (12)	0.0465 (11)	0.0401 (11)	0.0038 (10)	−0.0003 (10)	−0.0054 (9)
C6	0.0342 (10)	0.0439 (11)	0.0435 (11)	0.0035 (8)	0.0025 (10)	0.0006 (9)
C7	0.0474 (12)	0.0489 (12)	0.0517 (13)	0.0047 (11)	−0.0082 (12)	0.0020 (10)
C2	0.0342 (10)	0.0509 (12)	0.0490 (12)	−0.0003 (9)	0.0086 (10)	−0.0012 (10)
C10	0.0438 (12)	0.0505 (12)	0.0454 (12)	0.0058 (10)	0.0058 (11)	−0.0035 (10)
C11	0.0433 (12)	0.0619 (14)	0.0423 (12)	0.0026 (11)	0.0017 (10)	0.0014 (10)
C8	0.0682 (16)	0.0590 (14)	0.0411 (12)	0.0024 (13)	−0.0041 (13)	0.0023 (11)
C9	0.0633 (15)	0.0595 (14)	0.0414 (12)	0.0015 (13)	0.0100 (12)	−0.0025 (11)
C13	0.0472 (12)	0.0702 (16)	0.0542 (14)	−0.0016 (12)	−0.0033 (12)	−0.0035 (12)
C17	0.0600 (14)	0.0602 (15)	0.0470 (13)	−0.0119 (12)	−0.0011 (13)	−0.0033 (11)
C16	0.085 (2)	0.0626 (16)	0.0473 (14)	−0.0058 (16)	0.0077 (15)	0.0040 (12)
C14	0.0624 (17)	0.099 (2)	0.0545 (16)	0.0074 (17)	−0.0178 (15)	−0.0066 (16)
C15	0.092 (2)	0.0753 (19)	0.0444 (13)	0.0178 (18)	−0.0101 (16)	0.0030 (13)

### Geometric parameters (Å, º)

**Table:**

O1—C1	1.370 (2)	C2—H2	0.9300
O1—C11	1.423 (3)	C10—C9	1.370 (3)
C1—C6	1.368 (3)	C10—H10	0.9300
C1—C2	1.421 (3)	C11—H11A	0.9700
C5—C10	1.413 (3)	C11—H11B	0.9700
C5—C4	1.417 (3)	C8—C9	1.399 (4)
C5—C6	1.420 (3)	C8—H8	0.9300
C3—C2	1.354 (3)	C9—H9	0.9300
C3—C4	1.414 (3)	C13—C14	1.387 (4)
C3—H3	0.9300	C13—H13	0.9300
C4—C7	1.416 (3)	C17—C16	1.390 (4)
C12—C17	1.374 (3)	C17—H17	0.9300
C12—C13	1.377 (3)	C16—C15	1.367 (4)
C12—C11	1.499 (3)	C16—H16	0.9300
C6—H6	0.9300	C14—C15	1.375 (4)
C7—C8	1.361 (4)	C14—H14	0.9300
C7—H7	0.9300	C15—H15	0.9300
			
C1—O1—C11	116.36 (18)	O1—C11—C12	109.85 (19)
C6—C1—O1	125.7 (2)	O1—C11—H11A	109.7
C6—C1—C2	120.2 (2)	C12—C11—H11A	109.7
O1—C1—C2	114.1 (2)	O1—C11—H11B	109.7
C10—C5—C4	118.4 (2)	C12—C11—H11B	109.7
C10—C5—C6	122.0 (2)	H11A—C11—H11B	108.2
C4—C5—C6	119.61 (19)	C7—C8—C9	120.4 (2)
C2—C3—C4	121.3 (2)	C7—C8—H8	119.8
C2—C3—H3	119.4	C9—C8—H8	119.8
C4—C3—H3	119.4	C10—C9—C8	120.4 (2)
C3—C4—C7	122.2 (2)	C10—C9—H9	119.8
C3—C4—C5	118.47 (19)	C8—C9—H9	119.8
C7—C4—C5	119.3 (2)	C12—C13—C14	120.7 (3)
C17—C12—C13	118.9 (2)	C12—C13—H13	119.7
C17—C12—C11	121.5 (2)	C14—C13—H13	119.7
C13—C12—C11	119.5 (2)	C12—C17—C16	120.8 (3)
C1—C6—C5	120.1 (2)	C12—C17—H17	119.6
C1—C6—H6	120.0	C16—C17—H17	119.6
C5—C6—H6	120.0	C15—C16—C17	119.8 (3)
C8—C7—C4	120.6 (2)	C15—C16—H16	120.1
C8—C7—H7	119.7	C17—C16—H16	120.1
C4—C7—H7	119.7	C15—C14—C13	119.7 (3)
C3—C2—C1	120.3 (2)	C15—C14—H14	120.1
C3—C2—H2	119.8	C13—C14—H14	120.1
C1—C2—H2	119.8	C16—C15—C14	120.2 (3)
C9—C10—C5	120.9 (2)	C16—C15—H15	119.9
C9—C10—H10	119.5	C14—C15—H15	119.9
C5—C10—H10	119.5		
			
C11—O1—C1—C6	−5.6 (3)	C4—C5—C10—C9	−1.2 (3)
C11—O1—C1—C2	174.04 (19)	C6—C5—C10—C9	176.9 (2)
C2—C3—C4—C7	175.9 (2)	C1—O1—C11—C12	178.72 (19)
C2—C3—C4—C5	−2.3 (3)	C17—C12—C11—O1	−44.9 (3)
C10—C5—C4—C3	178.9 (2)	C13—C12—C11—O1	139.5 (2)
C6—C5—C4—C3	0.8 (3)	C4—C7—C8—C9	−0.3 (4)
C10—C5—C4—C7	0.8 (3)	C5—C10—C9—C8	0.9 (4)
C6—C5—C4—C7	−177.4 (2)	C7—C8—C9—C10	−0.1 (4)
O1—C1—C6—C5	177.4 (2)	C17—C12—C13—C14	−0.8 (4)
C2—C1—C6—C5	−2.2 (3)	C11—C12—C13—C14	174.9 (3)
C10—C5—C6—C1	−176.7 (2)	C13—C12—C17—C16	1.4 (4)
C4—C5—C6—C1	1.4 (3)	C11—C12—C17—C16	−174.2 (2)
C3—C4—C7—C8	−178.1 (2)	C12—C17—C16—C15	−0.7 (4)
C5—C4—C7—C8	0.0 (3)	C12—C13—C14—C15	−0.4 (4)
C4—C3—C2—C1	1.6 (3)	C17—C16—C15—C14	−0.6 (5)
C6—C1—C2—C3	0.7 (3)	C13—C14—C15—C16	1.2 (5)
O1—C1—C2—C3	−178.9 (2)		

### Hydrogen-bond geometry (Å, º)

*Cg*1, *Cg*2 and *Cg*3 are the centroids of the C1–C6, C4/C5/C7–C10 and C12–C17 rings, respectively.

**Table:**

*D*—H···*A*	*D*—H	H···*A*	*D*···*A*	*D*—H···*A*
C3—H3···*Cg*1^i^	0.93	2.71	3.439 (2)	135
C11—H11*A*···*Cg*2^ii^	0.97	2.63	3.512 (3)	150
C16—H16···*Cg*3^iii^	0.93	2.87	3.586 (3)	135

Symmetry codes: (i) *x*+1/2, −*y*+1/2, −*z*+1; (ii) *x*−1/2, −*y*+3/2, −*z*+1; (iii) −*x*+1, *y*+1/2, −*z*+1/2.
